# Oral *Candida* spp. Colonisation, the Immunoexpression of Selected Cytokines in Oral Mucosa Cells and Vitamin D3 Serum Levels in Patients with Different Severities of Psoriasis: A Case–Control Study

**DOI:** 10.3390/nu18172794

**Published:** 2026-08-26

**Authors:** Mariola Marchlewicz, Paulina Sagan, Marta Grabowska, Magdalena Kiedrowicz, Magdalena Boer, Joanna Kruk, Małgorzata Piasecka, Ewa Duchnik

**Affiliations:** 1 Department of Dermatology and Venereology, Faculty of Health Sciences, Pomeranian Medical University in Szczecin, 70-010 Police, Poland; mariola.marchlewicz@pum.edu.pl (M.M.); wietrakp@wp.pl (P.S.); magdalena.kiedrowicz@pum.edu.pl (M.K.); magdalena.boer@pum.edu.pl (M.B.); 2 Department of Histology and Developmental Biology, Faculty of Health Sciences, Pomeranian Medical University in Szczecin, 71-210 Szczecin, Poland; malgorzata.piasecka@pum.edu.pl; 3 Faculty of Physical Culture and Health, University of Szczecin, Piastów 40b/6, 71-065 Szczecin, Poland; joanna.kruk@usz.edu.pl; 4 Department of Aesthetic Dermatology, Faculty of Health Sciences, Pomeranian Medical University in Szczecin, Powstańców Wielkopolskich 72, 70-111 Szczecin, Poland; ewa.duchnik@pum.edu.pl

**Keywords:** psoriasis, *Candida* spp., oral mucosa, colonisation, vitamin D_3_ serum level, proinflammatory cytokines expression

## Abstract

**Background**: Psoriasis is the most common inflammatory skin disease, driven by cytokines including IL-12, IL-17, and TNF-α. Patients are frequently colonised with *Candida* spp., while vitamin D_3_ deficiency has been implicated in psoriasis pathogenesis and severity. This study compared oral *Candida* colonisation in patients with psoriasis and healthy controls, evaluated the relationship between vitamin D_3_ levels and disease severity, and assessed IL-12, IL-17, and TNF-α expression in oral mucosal cells. **Methods**: Fifty-nine patients with psoriasis and 32 healthy controls were enrolled. Psoriasis severity was assessed using the Psoriasis Area and Severity Index (PASI) and Body Surface Area (BSA). Oral swabs were collected for *Candida* spp. culture, and serum vitamin D_3_ levels were measured. Cytokine expression in oral mucosal cells was evaluated by immunohistochemistry and quantified using digital image analysis. **Results**: Lower serum vitamin D_3_ levels were associated with greater psoriasis severity. Oral mucosal colonisation by *Candida* spp. did not differ between patients and controls and was not associated with vitamin D_3_ levels or psoriasis severity. IL-12 and IL-17 expression was increased in patients with psoriasis compared with controls. IL-12 expression was higher in severe than mild psoriasis, while TNF-α expression was elevated in patients with more severe disease. **Conclusions**: In this cohort, no significant association was observed between oral *Candida* colonisation and psoriasis severity or the local cytokine response. In contrast, vitamin D_3_ deficiency was associated with more severe disease. Increased oral mucosal expression of IL-12, IL-17, and TNF-α indicates the presence of a local inflammatory response within the oral mucosa, which may be consistent with the concept of psoriasis as an inflammatory disorder extending beyond the skin.

## 1. Introduction

Psoriasis is a chronic inflammatory skin disease which has a complex pathogenesis and varies in severity. Chronic inflammation associated with psoriasis may contribute to the development of systemic complications [1]. Increased cardiovascular risk may contribute to poor quality of life and increased mortality. Studies have indicated the impact of vitamin D_3_ deficiency on the pathogenesis of psoriasis and its severity [2,3]. However, there is no clear evidence of the influence of the skin and mucosal candidiasis on the onset and progression of psoriasis.

Psoriasis is a dermatosis in which pathophysiology is fundamentally driven by a cytokine storm [4,5]. Proinflammatory interleukin 17 (IL-17), which is secreted mainly by Th-17 cells, plays a key role in this process. The effector cells for IL-17 are keratinocytes, neutrophils, macrophages, epithelial and endothelial cells, fibroblasts, osteoclasts, and chondrocytes. IL-17 stimulates the secretion of antimicrobial peptides, proinflammatory cytokines and chemokines, and normally functions in host defence against fungal infections. Therefore, IL-17 inhibitors increase the risk of fungal infections because in patients with psoriasis IL-17 plays an important role in a more intensified abnormal inflammation.

Clinical evidence of the role of IL-17, interleukin 12 (IL-12) and tumour necrosis factor alpha (TNF-α) in the development of psoriasis is the high effectiveness of TNF-α inhibitors, IL-17 and drugs blocking interleukins IL-12/IL-23 [6,7].

The role of TNF-α in the pathogenesis of psoriasis appears to be more indirect. TNF-α is secreted by T cells and antigen-presenting cells, but alone does not induce an inflammatory reaction in keratinocytes. However, TNF-α enhances the response to IL-17, and together they produce a synergistic effect by stabilising mRNA for IL-17A inhibitors. IL-12, on the other hand, is involved in redirecting the immune response towards enhanced Th1 cell activity and interferon gamma (IFN-γ) secretion [8].

Among the numerous yeast-like fungal species, *Candida* albicans is the most common commensal found in the mucous membranes of the human gastrointestinal tract. Epidemiological data indicate that up to 80% of the population has their oral cavity colonised by C. albicans [9]. However, in immunocompromised patients, it may cause opportunistic mucosal infections [10]. The pathogenic potential of C. albicans relies strongly on its ability to undergo a morphological transition from unicellular yeasts (blastospores) to invasive hyphae [11]. The invasive form shows adherence to epithelial cells and thigmotropic behaviour [9]. In the state of microbial–immune balance, the immune system does not respond to the presence of non-invasive forms of *Candida* spp. It is unclear why the full immune response is triggered by the progressing infection. One explanation may come from the fact that the cell membrane of the hyphal form of C. albicans contains more immunogenic oligosaccharides, which are involved in the stimulation of human immune cells [12].

Recent studies have provided new, though still inconsistent, evidence on the association between *Candida* colonisation and psoriasis [13,14,15,16,17]. In a large case–control study Elsner et al. [15] found a significantly higher prevalence of oral *Candida* colonisation in patients with psoriasis than in controls; however, no significant relationship was observed between the presence of *Candida* and disease severity or the systemic therapy used. These findings were partly corroborated by a systematic review and trial sequential analysis by Patini et al. [16], which demonstrated that patients with psoriasis have a more than three-fold higher odds of oral *Candida* colonisation compared with individuals without psoriasis. More recently, Campione et al. [17] reported *Candida* spp. colonisation in 47% of patients with psoriasis involving difficult-to-treat areas, with the oral cavity affected in 34% of the study group.

There are many hypotheses on the relationship between *Candida* spp. colonisation and the immune response in patients with psoriasis. Experimental studies have demonstrated that *Candida* spp. can act as a superantigen, non-specifically stimulating T cells to produce IL-17, and can activate Neutrophil Extracellular Traps (NETs) [18,19]. These findings have inspired some researchers to hypothesise that *Candida* spp. may be involved in the pathogenesis of psoriasis. This hypothesis is indirectly supported by the recent observations of Campione et al. [17]. The authors suggested that fungi may participate in the pathogenesis of psoriasis, among others via activation of the IL-17/IL-23 axis, although their results do not prove a causal relationship.

Psoriasis patients are at risk of hypovitaminosis D_3_, which may indicate a role of vitamin D_3_ deficiency in the pathogenesis of psoriasis [2,20,21]. Vitamin D_3_ is the most bioavailable form of vitamin D. It is produced in the skin under the influence of ultraviolet B (UVB) light and is present in animal-sourced foods. Vitamin D supports many important body functions, including the skin, mucous membranes, and the immune system, for example by influencing the differentiation and activity of keratinocytes, increasing the synthesis of keratins 1 and 10, regulating apoptosis, reducing the proliferation and differentiation of T and B cells, stimulating the expression of antimicrobial peptides (AMPs), and exhibiting significant antioxidant activity [20,22,23]. The immunomodulating effect of vitamin D_3_ and the normalisation of keratinocytes may contribute to this relationship. The mechanisms responsible for vitamin D_3_’s protective effects against an inadequate immune response and the onset of autoimmune diseases such as type 1 diabetes, multiple sclerosis, inflammatory bowel disease, rheumatoid arthritis, skin diseases, and thyroid diseases include the suppressed expression of toll-like receptors (TLRs) on dendritic cells, which results in reduced stimulation of Th1 and Th17 cells to secrete proinflammatory cytokines [8,24,25].

Evidence indicates that a Body Mass Index (BMI) higher than 27 kg/m^2^ (BMI > 27) is an additional risk factor for vitamin D_3_ deficiency in psoriasis patients [21]. Despite evidence on the role of vitamin D_3_ in the physiology of the skin, no supplementation guidelines have been established for patients with psoriasis [20].

Given the conflicting results of recent studies on the prevalence and clinical significance of oral *Candida* colonisation in patients with psoriasis [15,16,17], the aim of this study was to investigate oral *Candida* colonisation and serum levels of vitamin D_3_ in patients with psoriasis vulgaris and to assess their association with disease severity. An attempt was also made to assess the immunoexpression of selected proinflammatory cytokines in the oral mucosa of patients with psoriasis vulgaris and in controls.

## 2. Materials and Methods

### 2.1. Study Design

This case–control study involved 91 individuals aged 23–85 years (38 women and 53 men) hospitalised in the Department and Clinic of Skin and Venereal Diseases and being under the care of the Outpatient Clinic of Skin and Venereal Diseases of the Pomeranian Medical University in Szczecin, Poland. All patients had a physical examination, with particular focus on assessing the severity of psoriasis using the Psoriasis Area and Severity Index (PASI) and Body Surface Area Index (BSA). The control group included individuals without skin conditions (*n* = 32), while the study group consisted of patients with psoriasis (*n* = 59). Depending on the severity of psoriasis, patients were divided into two subgroups: mild psoriasis (PASI scores < 10, BSA scores < 10) and moderate to severe psoriasis (PASI scores ≥ 10, BSA scores ≥ 10). Each subject underwent a thorough questionnaire-based interview, covering socioeconomic status, lifestyle, the course of the primary disease, comorbidities, and treatment. Nutritional status was assessed using BMI for each subject.

This study was approved by the Bioethics Committee of the Pomeranian Medical University in Szczecin (approval no. KB-0012/30/19). The methodology and purpose of the study was explained to all subjects, and they gave written informed consent to participate in the study.

### 2.2. Selection Criteria

We included subjects who had a clinical diagnosis of psoriasis, were aged 18 to 85 years, and gave written informed consent to participate in the study.

We excluded patients who:

- Used immunosuppressants (including biologicals and phototherapy) within previous 12 weeks;
- Used antifungals within previous 12 weeks;
- Used antibiotics within previous 12 weeks;
- Had a history of skin disease other than psoriasis;
- Had an infectious disease (bacterial, fungal, viral, including COVID-19) within previous 2 weeks;
- Had a history of renal or hepatic failure;
- Had a history of hypoparathyroidism;
- Were unprepared for clinical tests, reported for the examination after having a meal and/or doing morning oral hygiene.

### 2.3. Biochemical Assays

Blood samples were collected from patients who had been fasting for at least 10 h. Serum 25-hydroxyvitamin D [25(OH)D] concentrations were determined using a chemiluminescent immunoassay (CLIA) performed on a LIAISON XL automated analyzer (DiaSorin, Saluggia, Italy) with the LIAISON 25 OH Vitamin D Total Assay 200 reagent kit (ref. no. 318360; DiaSorin, Saluggia, Italy). Vitamin D status was classified as deficient/low (<20 ng/mL), suboptimal (20–30 ng/mL), optimal (>30–50 ng/mL), high (>50–100 ng/mL), or potentially toxic (>100 ng/mL).

### 2.4. Mycological Assays

Mycological assays of swabs collected from the oral cavity were performed for all subjects. Culture material was collected using swab kits supplied in a plastic tube, with a ca. 15 cm long shaft and a viscose tip ca. 5 mm in diameter, without medium. Swabs were collected from the oral mucosa of patients who had not eaten, drunk, brushed their teeth, or used mouthwash for at least 10 h before the procedure.

The obtained culture material was applied to a Petri dish with Sabouraud medium (BTL spółka z o.o. Zakład Enzymów i Peptonów, Łódź, Poland) with the addition of chloramphenicol (Sigma-Aldrich, St. Louis, MO, USA). Sabouraud medium contained: distilled water, agar, glucose and peptones; it is a selective medium for the isolation of various types of yeasts and filamentous fungi. The addition of chloramphenicol inhibited the growth of most Gram (+) and Gram (−) bacteria. Petri dishes containing agar were stored at 3 °C prior to the assay. Before the material was cultured, Petri dishes were warmed to room temperature 20–22 °C. Cultures on Sabouraud medium were incubated for 72 h in an incubator at 36 °C ± 1 °C and then at room temperature for 10 days, and inspected for growth every 2 days. A positive culture was considered to be the growth of typical macroscopic yeast-like colonies of *Candida* spp., which are round, smooth, convex, clearly demarcated, white, and cause no change in medium colour.

*Candida* spp. were identified using the colorimetric AuxaColor 2 Kit (Bio-Rad Laboratories Inc., Hercules, CA, USA). This identification system is based on the principle of sugar assimilation, and the growth of yeasts is visualised by the colour change of a pH indicator. The kit comprises a negative control (without sugars) and 13 sugar assimilation results corresponding to the following sugars: glucose (positive control), maltose, cellobiose, sucrose, trehalose, galactose, adonitol, lactose, melezitose, raffinose, xylose, inositol, and arabinose. Sugar assimilation and the growth of a yeast are indicated by the colour change of the indicator from blue to yellow and by a cloudy appearance in the well.

Following the manual supplied with the AuxaColor 2 Kit, a suspension of R2 reagent was inoculated with 24 to 48 h cultures of the tested strain colonies (opacity equal to 1.5 McFarland scale (MCF). Inoculum (100 µL) was collected and distributed to each well of the microplate (R1). The microplate was covered with adhesive film, ensuring perfectly uniform adhesion, and incubated for 48 to 72 h at 30 °C (±2 °C). Yeast species were identified by the colour change of the indicator in the wells indicating biochemical reactions.

### 2.5. Immunohistochemical Assay

The study included non-smoking patients without symptoms of periodontitis and without a history of a symptomatic upper respiratory tract infection within the previous month. The immunoexpression of cytokines IL-12, IL-17, and TNF-α was detected by the immunohistochemical assay. Cytology material was collected from the oral mucosa of 15 randomly selected individuals from each study group using soft-bristle, fan-shaped, plastic cytology swabs supplied in single packages.

Diagnostic material collected from the oral mucosa of patients who had not eaten, drunk, brushed their teeth, or used mouthwash for at least 10 h before the swab was spread on adhesive glass slides and left for 15 min to dry. The prepared microscopic slide was fixed in 96% ethyl alcohol and placed in a 0.9% NaCl solution.

Antigen retrieval was performed by boiling slides with oral epithelial cells for 30 min in citrate buffer (Dako, Glostrup, Denmark) at pH 6.0 (for identification of IL-12 and IL-17) and in pH 9.0 buffer (for TNF-α). Endogenous peroxidase was blocked using Peroxidase Blocking Solution (Dako, Glostrup, Denmark) for 10 min at room temperature. The immunoexpression of specific proteins was identified using the following polyclonal antibodies (Nordic BioSite, Taby, Sweden), diluted 1:200: (1) mouse anti-TNF-α; (2) rabbit anti-IL-12 and anti-IL-17. Slides with oral epithelial cells were incubated with primary antibodies in a humid chamber for 30 min and then with a complex reagent containing horseradish peroxidase conjugated secondary antibodies (Dako, Glostrup, Denmark). Diaminobenzidine (Dako, Glostrup, Denmark) was used to visualise the immunohistochemical staining. All slides were counterstained with Mayer’s haematoxilin (Sigma-Aldrich Co., St. Louis, MO, USA), dehydrated, and coverslipped. Slides were analysed under a light microscope (Olympus BX 41, Hamburg, Germany). The specificity of the reaction was also verified.

Oral epithelial cells used in immunohistochemical assays were scanned at 400× magnification using a ScanScope AT2 histology slide scanner (Leica Microsystems, Wetzlar, Germany). The acquired digital images of epithelial cells were analysed on a computer screen using ImageScope Viewer software (version 11.2.0.780; Aperio Technologies, Vista, CA, USA). The immunoexpression of TNFα, IL12, and IL17 was quantified on slides using the cytoplasmic v9 algorithm (Aperio Technologies, Vista, CA, USA). All parameters were set to achieve consistency with the visual assessment of colour intensity. The percentage of positive immunostaining for each marker and for each patient group was determined in 30 fields with an average area of 18.7 mm^2^ (for TNF-α), 18.5 mm^2^ (for IL-12), or 19.6 mm^2^ (for IL-17).

### 2.6. Statistical Analysis

The obtained data were analysed using the IBM SPSS Statistics package, version 27. Continuous variables were presented as means (M) and standard deviations (SD). The distribution of variables for normality was analysed using the Shapiro–Wilk test, and the levels of skewness and kurtosis were assessed. Following Hair et al. [26], data are considered to be normally distributed if the absolute skewness is between −2 and +2 and the absolute kurtosis is between −7 and +7. Vitamin D_3_ serum levels were assessed with the one-way ANOVA analysis of covariance using the parametric F-test for variables with a near-normal distribution, and considering BMI as a confounding variable. Data on the colonisation of the oral mucosa with *Candida* spp. were checked for statistical significance using the chi-square test. Results of immunohistochemical assays were assessed using the non-parametric Kruskal–Wallis test for non-normally distributed variables.

Two-stage statistical analyses were performed to determine the relationship between two continuous variables. In the first stage, Pearson’s r was calculated for variables with a near-normal distribution, and the non-parametric Spearman’s rho correlation coefficient was calculated for variables with a non-normal distribution. In the second stage, if a significant statistical correlation was found between variables, a stepwise multivariate regression analysis was performed to determine the most significant predictor for the explained variable [27]. The chi-square cross-tabulation test was used to determine the relationship between two categorical variables. In all analyses, the level of statistical significance was adopted at α ≤ 0.05.

## 3. Results

### 3.1. Characteristics of Demographic and Clinical Variables

The statistical analysis did not reveal any significant differences between patients with psoriasis and the control group in terms of age (*t* = 0.08; *p* = 0.938), body weight (*t* = 0.73; *p* = 0.470), body height (*t* = 1.37; *p* = 0.173), BMI (*t* = 0.32; *p* = 0.752) or the period of vitamin D_3_ intake (χ^2^ = 0.04; *p* = 0.841 (Table 1).

Analysed groups differed significantly in terms of gender (χ^2^ = 8.73; *p* = 0.003): in the group of patients with psoriasis, there were more men than women (41 and 18, respectively, *p* = 0.003), and in the control group, there were more women than men (20 and 12, respectively, (*p* = 0.003).

No significant differences were found between patients with psoriasis regardless of its severity and controls in terms of age (*F* = 0.47; *p* = 0.628), body weight (*F* = 0.27; *p* = 0.764), body height (*F* = 2.96, *p* = 0.057), BMI score (*F* = 0.28, *p* = 0.753), and the period of vitamin D_3_ intake (χ^2^ = 0,04, *p* = 0.979) (Table 2).

However, the study groups differed significantly in terms of gender (χ^2^ = 14.84, *p* < 0.001): there were more men than women in the subgroup of patients with moderate to severe psoriasis (22 and 3, respectively, *p* < 0.001); and there were more women than men in the control group (20 and 12, respectively, *p* = 0.003).

There were no significant differences between the subgroup of psoriasis patients with low vitamin D_3_ levels, those with suboptimal vitamin D_3_ levels, or the subgroups with optimal and high vitamin D_3_ levels in terms of body weight (*F* = 0.60, *p* = 0.555), BMI (*F* = 0.42, *p* = 0.662), and period of vitamin D_3_ intake (χ^2^ = 0.98, *p* = 0.614) (Table 3). However, the study groups differed significantly in terms of age (*F* = 3.25, *p* = 0.046), body height (*F* = 6.93, *p* = 0.002), and gender (χ^2^ = 7.57, *p* = 0.023). In the group of psoriasis patients with suboptimal vitamin D_3_ levels, there were more women than men (8 and 5, respectively; *p* = 0.005).

### 3.2. Oral Mucosa Colonisation by Candida spp. in Patients with Psoriasis and Controls

Findings on the colonisation of the oral mucosa with *Candida* spp. in the analysed groups are summarised in Table 4. Statistical analysis of data on the colonisation of the oral mucosa by *Candida* spp. did not reveal any significant differences between patients with psoriasis and controls (χ^2^ = 0.01, *p* = 0.938).

No significant differences were also found in the prevalence of *Candida* colonisation between patients with moderate to severe psoriasis and patients with mild psoriasis (χ^2^ = 0.02; *p* = 0.879).

### 3.3. Effect of Vitamin D_3_ Level on the Severity of Psoriasis

There were no significant differences in the severity of psoriasis between patients with low vitamin D_3_ levels, patients with suboptimal vitamin D_3_ levels, and patients with optimal or high vitamin D_3_ levels (Table 5). However, the analysis did reveal a trend toward a positive effect of higher vitamin D_3_ levels: mild psoriasis was found in 19 patients with suboptimal, optimal and high vitamin D_3_ levels, while moderate to severe psoriasis was diagnosed in eight patients from these subgroups (χ^2^ = 5.39; *p* = 0.067).

Additional comparative analysis showed that mild psoriasis was diagnosed more frequently than moderate to severe psoriasis in the subgroup of patients with optimal or high vitamin D_3_ levels (*p* = 0.030).

The statistical significance of differences between patients with different severities of psoriasis and vitamin D_3_ levels was assessed using PASI and BSA scores (Table 6). PASI scores showed that patients with low vitamin D_3_ levels, suboptimal vitamin D_3_ levels, and optimal or high vitamin D_3_ levels differed in terms of disease severity, and a statistical trend was observed (*F* = 3.10, *p* = 0.053). The effect size for this parameter was medium (ɳ^2^ = 0.10). BMI was an insignificant confounding variable for the differences in disease severity assessed with PASI (*F* = 0.10, *p* = 0.748).

A slightly stronger effect of vitamin D_3_ level on disease severity was observed when BSA score was used for assessment. Patients with psoriasis and low, suboptimal, and optimal or high vitamin D_3_ levels differed significantly in terms of disease severity as a continuous variable (*F* = 3.84; *p* = 0.028). The effect size of differences for this variable was medium (*ɳ*^2^ = 0.13). BMI was an insignificant confounding variable for the differences in psoriasis severity measured with BSA in the analysed groups (*F* = 0.11; *p* = 0.740).

### 3.4. Effect of Vitamin D_3_ Levels on Oral Candida Colonisation in Patients with Psoriasis and Controls

Statistical analysis showed no significant differences between psoriatic patients with low, suboptimal, and optimal or high vitamin D_3_ levels in terms of colonisation of the oral mucosa with yeast-like *Candida* spp. (χ^2^ = 3.76; *p* = 0.153) (Table 7).

Similarly, there were no significant differences in the control group between subjects with low vitamin D_3_ levels, suboptimal vitamin D_3_ levels, and with optimal or high vitamin D_3_ levels in terms of colonisation of the oral mucosa by *Candida* spp. (χ^2^ = 2.26; *p* = 0.323).

### 3.5. Effect of Vitamin D_3_ Serum Levels and Oral Candida Colonisation on the Severity of Psoriasis

Statistics on the effect of different vitamin D_3_ levels and oral *Candida* colonisation on the severity of psoriasis measured with PASI or BSA scores are presented in Table 8 and Table 9, respectively. For PASI, psoriasis patients with low vitamin D_3_ levels, patients with suboptimal vitamin D_3_ levels, and patients with optimal or high vitamin D_3_ levels differed in disease severity (assessed as a continuous variable), and a statistical trend was observed (*F* = 3.14, *p* = 0.051) (Table 8). The effect size of differences for this parameter was medium (*ɳ*^2^ = 0.11). No significant differences were found between patients with and without oral *Candida* colonisation in terms of psoriasis severity (*F* = 0.24, *p* = 0.628, *ɳ*^2^ = 0.00). There was also no interaction between the between-group factors, i.e., vitamin D_3_ level and positive or negative *Candida* culture, and the severity of psoriasis (*F* = 0.02, *p* = 0.993, ɳ^2^ = 0.00).

Post hoc analysis revealed that patients with low vitamin D_3_ levels had more severe psoriasis (a continuous variable measured with PASI) compared to psoriasis patients with suboptimal vitamin D_3_ levels (*p* = 0.047).

Table 9 presents the effect of vitamin D_3_ levels on the severity of psoriasis measured with BSA in patients with and without oral *Candida* colonisation. The severity of psoriasis in patients with low vitamin D_3_ levels, suboptimal vitamin D_3_ levels, and optimal or high vitamin D_3_ levels differed significantly (*F* = 4.09, *p* = 0.022). The effect size of differences for this parameter was medium (*ɳ*^2^ = 0.13). However, no differences were found in the severity of psoriasis between patients with and without oral *Candida* colonisation (*F* = 0.31, *p* = 0.582). The effect size of differences for this parameter was negligible (*ɳ*^2^ < 0.01). There was also no interaction effect between the between-group factors, i.e., vitamin D_3_ level and positive or negative *Candida* culture, and the severity of psoriasis (*F* = 0.25, *p* = 0.779). The effect size of differences for this parameter was negligible (*ɳ*^2^ < 0.01).

Post hoc analysis revealed that psoriasis patients with low vitamin D_3_ levels had more severe disease (a continuous variable measured with the BSA score) compared to psoriasis patients with suboptimal vitamin D_3_ levels (*p* = 0.018).

### 3.6. Immunoexpression of IL-12, IL-17 and TNF-α in Oral Epithelial Cells

**Figure 1:**
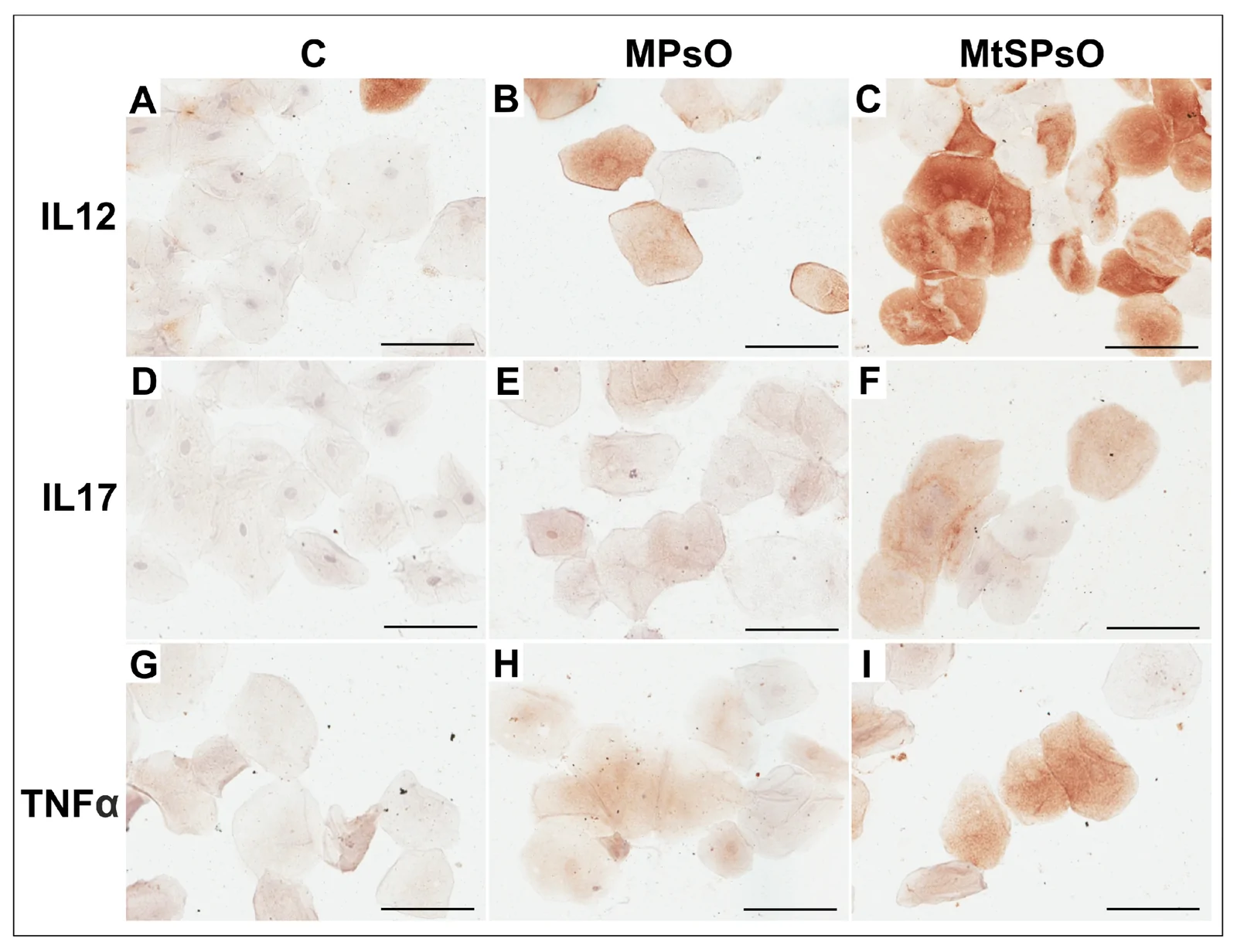
Representative photomicrographs showing the immunoexpression (brown cytoplasm) of IL-12 (panels (**A**–**C**)), IL-17 (panels (**D**–**F**)) and TNF-α (panels (**G**–**I**)) in oral epithelial cells obtained from control subjects (**A**,**D**,**G**), patients with mild psoriasis (**B**,**E**,**H**) and patients with moderate to severe psoriasis (**C**,**F**,**I**). Scale bar—100 µm. Abbreviations: IL-12—interleukin 12; IL-17—interleukin 17; TNF-α—tumour necrosis factor α; C—controls; MPsO—patients with mild psoriasis; MtSPsO—patients with moderate to severe psoriasis.

The immunoexpression of cytokines TNFα, IL12, and IL17 in oral epithelial cells obtained from control subjects and patients with mild or moderate to severe psoriasis was indicated by brown-stained cell cytoplasm (Figure 1).

**Figure 2:**
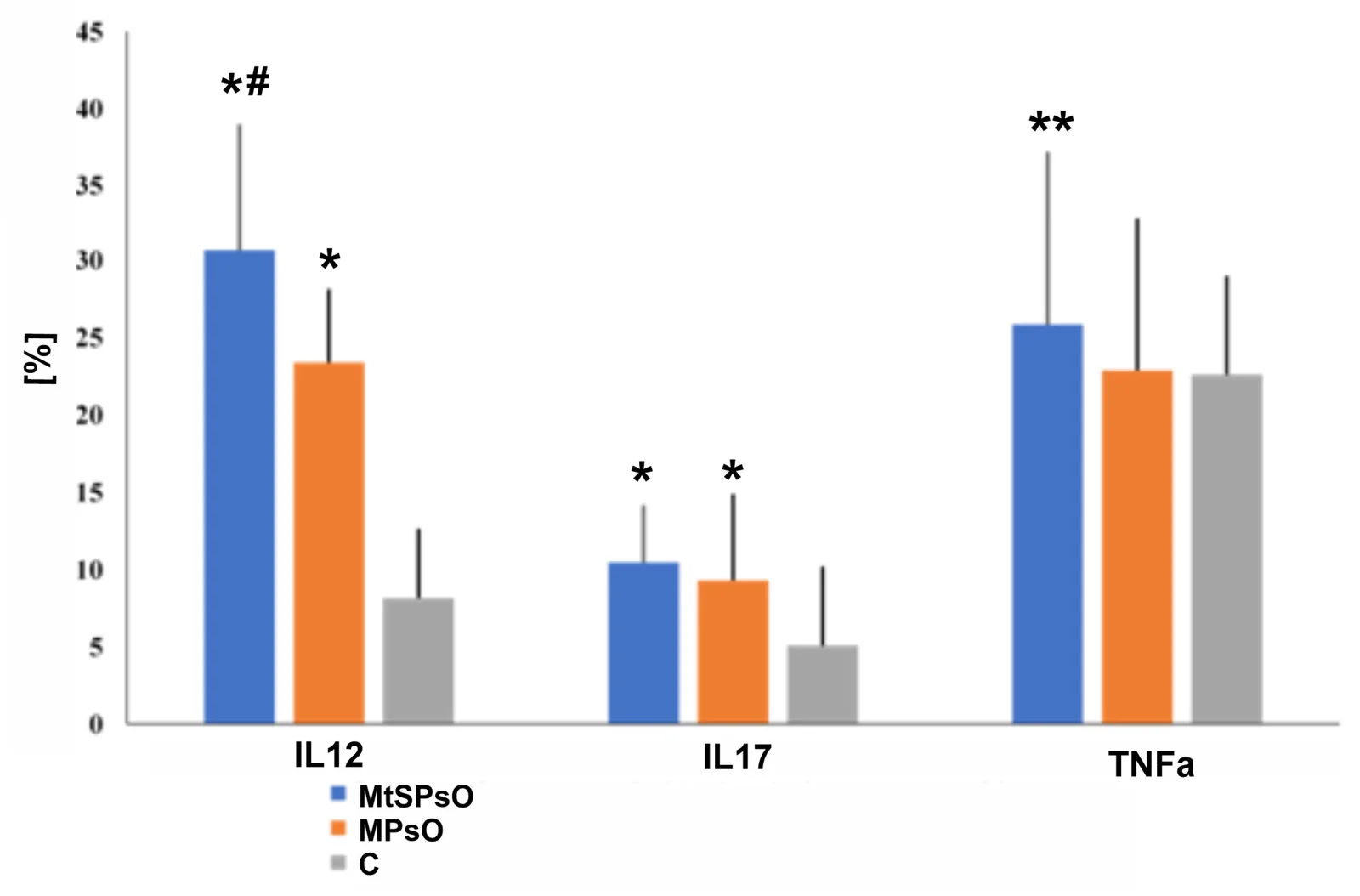
Percentage of oral epithelial cells expressing cytokines IL-12, IL-17 and TNF- α in patients with severe to moderate psoriasis (*n* = 15), in patients with mild psoriasis (*n* = 15) and in controls (*n* = 15). IL-12—interleukin 12; IL-17—interleukin 17; TNF-α—tumour necrosis factor α; C—controls; MPsO—patients with mild psoriasis; MtSPsO—patients with moderate to severe psoriasis; *—*p* < 0.001 vs. control; **—*p* < 0.05 vs. control; #—*p* < 0.005 vs. MPsO.

Quantitative analysis of the immunoexpression of these inflammatory markers revealed a significantly higher percentage of cells expressing these cytokines in patients with psoriasis, regardless of disease severity, compared to controls. Post hoc analysis revealed that the percentage of cells expressing IL-12 and IL-17 was significantly higher in patients with mild and moderate to severe psoriasis compared to controls (*p* < 0.001). Moreover, the percentage of IL-12-positive cells was significantly higher in patients with moderate to severe psoriasis than in patients with mild psoriasis (*p* = 0.028). The percentage of cells expressing TNF-α was significantly higher only in patients with moderate to severe psoriasis compared to controls (*p* = 0.003). The percentage of TNF-α-positive cells was higher in patients with mild psoriasis compared to controls, but statistical significance at the confidence level of *p* ≤ 0.05 was not found (Figure 2).

## 4. Discussion

This study analysed the prevalence of oral mucosa colonisation by *Candida* spp., the effect of serum vitamin D_3_ levels on the prevalence of psoriasis vulgaris, and the relationships between vitamin D_3_ levels, oral *Candida* colonisation and psoriasis severity. The immunoexpression of selected proinflammatory cytokines in the oral mucosa of psoriasis patients and in control subjects was also assessed.

The study included patients who had not received antipsoriatic treatment, which eliminated the influence of pharmacotherapy on the analysed variables. Parameters were assessed in the study and control groups at the same times of year to eliminate the effect of seasonality on serum vitamin D_3_ levels. Mycological assays were performed by the same investigator for all study participants. All patients were assessed using BSA and PASI by the same physician, who was blinded to the results of biochemical, immunohistochemical and mycological assays. This approach reduced the likelihood of bias and standardised the assessments with BSA and PASI, which are subjective in some respect.

The present study revealed no significant differences in the prevalence of oral *Candida* colonisation between patients with psoriasis and control subjects. Our results are in contrast to the findings by Leibovici et al. [28] and Senff et al. [29] and to the pooled estimate of a recent systematic review and trial sequential analysis by Patini et al. [16]. This may be due to at least several reasons. There are many factors influencing the colonisation of the oral cavity by *Candida* species. One of these is immunosuppressant treatment of psoriasis, which significantly increases the risk of fungal colonisation of the mucosa. For example, a significant increase in the prevalence of fungal infections was confirmed in patients with psoriasis during treatment with IL-17 inhibitors, secukinumab and ixekizumab [30]. Pietrzak et al. [13] also reported that the colonisation of the oral cavity by *Candida* spp. was significantly higher in patients receiving immunosuppressive therapy. In contrast to these data, our study investigated only patients who had not previously received immunosuppressants or immunomodulants. Therefore, it appears that mucosal colonisation by *Candida* spp. in patients with psoriasis is most likely secondary to systemic therapy.

Diabetes, dental caries, xerostomia, and wearing dentures also significantly increase the risk of oral *Candida* colonisation. Frequent hospitalizations have also been found to influence the onset of fungal infections [31,32]. Therefore, the coexistence of chronic diseases, extensive contact with the health care system, and socioeconomic status may be closely linked to the risk of fungal colonisation. Previously published studies did not consider these factors in the comparative analysis of patients with and without psoriasis, which could have significantly influenced the reported observations.

Also, the sampling technique used for mycological assays may influence the detection rate of oral mucosal colonisation. Positive *Candida* cultures are less frequently obtained from oral swabs compared to oral rinses with saline [33]. Although the above-mentioned studies used a uniform sampling technique in the compared groups, the sensitivity of the method may be important in patients with a low-grade *Candida* colonisation. The lack of statistically significant differences in the prevalence of oral *Candida* colonisation between patients with psoriasis and controls may also be due to the size of the analysed population. It should be noted, however, that the high probability value obtained (*p* = 0.938) reduces the likelihood that a clinically relevant difference was missed solely due to sample size. Our findings suggest that there are currently no clear data confirming a more frequent colonisation of the oral cavity with *Candida* spp. in patients with psoriasis who have not received therapy.

In the present study, no association was also identified in the studied cohort between oral *Candida* colonisation and the severity of psoriasis. Experimental and clinical studies conducted to date on this issue have provided contradictory results. For example, Nakajima et al. [34] demonstrated that skin exposure to *Candida* albicans exacerbates inflammation in a murine model of psoriasis by increasing the level of IL-17. Ovčina-Kurtović et al. [35] described an association between the presence of *Candida* spp. in skin folds and the severity of psoriasis evaluated with a PASI score. Contradictory results were reported by Sarvtin et al. [36], who found no significant correlation between *Candida* spp. colonisation or the presence of antibodies against *Candida* spp. and the clinical severity of psoriasis. Elsner et al. [15] also found no significant influence of *Candida* colonisation on the severity of psoriasis or levels of selected cytokines. Although some experimental and clinical studies indicate a potential effect of *Candida* colonisation on the severity of psoriasis, streptococci are a much more likely factor stimulating T cells through the mechanism of antigenic mimicry [37]. Moreover, some authors observed that the structure of T-cell receptors in chronic psoriasis is altered and is characterised by a lower susceptibility of T cells to superantigens [38]. Therefore, oral colonisation may not be a major determinant of psoriasis severity, which would be consistent with the lack of association observed in our cohort. However, this observation requires confirmation in larger studies using more sensitive detection methods.

Treatment with IL-17 inhibitors such as secukinumab, ixekizumab, and bimekizumab, is an independent risk factor for the development of fungal infections in patients with psoriasis [30]. The decrease in IL-17 levels observed during treatment is associated with a proportional clinical improvement evaluated with PASI. This suggests that the clinical impact of *Candida* colonisation, or even *Candida* infection, on psoriasis severity may be limited when IL-17 activity is inhibited, pointing primarily to the key role of IL-17 in psoriasis exacerbation. However, it also indirectly suggests the importance of gene polymorphisms for IL-17 and its receptor in psoriasis. Patients with comparable IL-17 levels may present different degrees of cellular response and, therefore, have a different clinical manifestation of psoriasis. For example, patients with a lower expression of the IL-17 receptor may not respond to *Candida* spp. infection with a significantly increased immune response.

At present, it should be concluded that despite preliminary data from experimental studies investigating the influence of *Candida* albicans colonisation on the severity of psoriasis, clinical evidence of such a relationship is still inconclusive and this aspect requires further research.

In the present study, only a statistical trend was observed for data on vitamin D_3_ levels and psoriasis severity measured with PASI. However, a significant correlation was found between vitamin D_3_ levels and BSA scores. The skin area affected by psoriatic lesions was significantly larger in patients with low vitamin D_3_ levels (<10 ng/mL) than in those with suboptimal vitamin D_3_ levels (20–30 ng/mL) when disease severity was assessed using BSA scores. In contrast, PASI analysis demonstrated only a statistical trend. Moreover, the categorical analysis of psoriasis severity did not reach statistical significance. These findings suggest that lower vitamin D_3_ levels may be associated with more severe disease. Nevertheless, the interpretation of this association should take into account the potential influence of demographic variables. Other factors may potentially influence the relationship between vitamin D_3_ status and psoriasis severity. Age may affect both serum 25(OH)D concentrations and the clinical course of psoriasis. In our study, patients with different vitamin D_3_ levels differed significantly in terms of age, suggesting that age-related factors could have partially contributed to the observed associations.

Our study does not clearly explain whether low vitamin D_3_ levels are a cause or a complication of the severe course of psoriasis. In patients with metabolic syndrome, cholecalciferol is sequestered in adipose tissue. Obesity is also an independent risk factor for a more severe course of psoriasis, which is facilitated by the secretion of IL-17 in adipocytes. It has also been emphasised that the severity of psoriasis independently contributes to reduced quality of life and the onset of metabolic syndrome, often resulting from a lower level of physical activity and giving up sports [20,39,40]. Therefore, a possible explanation for the relationship between the severity of psoriasis and low serum vitamin D levels is the coexistence of obesity [41]. In the present study, however, a covariance test including BMI as a covariate showed that it was not a confounding variable in the studied relationship. Therefore, the relationship between vitamin D_3_ level and psoriasis severity in the study group probably had a different pathogenesis and was not related to excess adipose tissue. In addition, sex should also be considered when interpreting the relationship between vitamin D_3_ status and psoriasis severity. It is worth mentioning that the sex distribution within the individual groups reflected the characteristics of patients who met the inclusion criteria during the recruitment period. In particular, a predominance of men was observed in the group of patients with severe psoriasis. This observation is consistent with previous epidemiological studies indicating that men may have greater objectively assessed psoriasis severity than women [42]. In a large study based on the Swedish PsoReg registry, including 5438 patients, PASI scores were significantly higher in men than in women [42]. Another study also demonstrated that the higher proportion of men among patients receiving biologic therapy may be at least partly attributable to greater disease severity in this group [43]. Thus, the predominance of men in the severe psoriasis group in our study may partly reflect the actual characteristics of the population of patients with more severe disease.

Sex-related differences may also be relevant in the context of vitamin D metabolism, immune regulation, and fungal colonisation. Sex may potentially influence vitamin D levels, immune responses, and *Candida* colonisation. However, it should be emphasised that the effect of sex on the parameters analysed in our study is not unequivocal, and significant sex-related differences have not been demonstrated consistently across studies. In the study by Mun et al., which included 203 asymptomatic individuals, no significant differences in the prevalence of oral *Candida* carriage were observed between women and men [44]. Similarly, Lippi et al., analysing 25(OH)D concentrations in 2327 individuals, found no significant differences between women and men either in 25(OH)D concentrations or in the prevalence of vitamin D deficiency [45].

These findings indicate that, although sex may be one of the factors influencing the parameters investigated, it may not necessarily represent a dominant determinant. Both vitamin D status and oral *Candida* colonisation are multifactorial and may depend on a range of clinical, environmental, and individual factors.

Another potential secondary factor that could contribute to lower vitamin D_3_ levels in patients with more severe psoriasis is the avoidance of sun exposure by patients with advanced skin lesions [41]. Despite the beneficial effect of ultraviolet light on psoriasis, the influence of psychological avoidance of the sun during the summer in patients with advanced skin lesions cannot be ruled out. It is also possible that psoriatic skin lesions impede vitamin D_3_ production due to damage to the epidermal layers and inflammation in the dermis. This could explain why the relationship between low vitamin D_3_ levels and BSA scores shown in our study was statistically stronger than that for the PASI scores.

Our findings partially support previous studies by Orgaz-Molina et al. [21] and Ricerri et al. [41], which revealed a negative correlation between PASI scores and vitamin D_3_ levels. These authors demonstrated the persistence of the identified correlation even after the elimination of the effects of sun exposure time, vitamin D_3_ supplementation, BMI, and Fitzpatrick skin type [46]. However, in our study, the relationship depended on the clinical assessment method. A statistically significant association was observed when disease severity was assessed using BSA, whereas only a statistical trend was found for PASI scores. These findings do not establish a causal relationship between vitamin D_3_ levels and psoriasis severity. Nevertheless, a potential role of vitamin D_3_ is biologically plausible, given its involvement in regulating local and systemic inflammatory responses and the association between vitamin D_3_ deficiency and dysregulated secretion of proinflammatory cytokines, including IL-6, IL-8, IL-1β, and TNF-α [20,21,22,23].

However, the above studies did not examine the effect of physical activity on vitamin D_3_ concentration. The existing scientific literature has well documented the positive impact of physical activity on physical and mental health, due to its multifactorial biological action [47,48]. Regular moderate-to-vigorous physical activity (3–<6 METs) decreases inflammation, lowers levels of the proinflammatory cytokines IL-6, IL-1β, and TNF-α, controls redox homeostasis, stimulates vitamin D release from adipose tissue, enhances immune cell function, reduces viral loads, body fat and oxidative stress, as well as regulates resistance to it (among others). In addition, endurance and resistance physical exercise can repair damaged mitochondria, increase their volume and activity, and enhance mitochondrial biogenesis by increasing PGC-α expression. Physical activity may affect skin diseases, including psoriasis, although the importance of physical activity in preventing and mitigating psoriasis has been underestimated by scientists relative to studies on diet [49]. The commonly proposed mechanisms that may explain the beneficial effects of physical activity on psoriasis involve: helping to keep a healthy body weight; reduction of body fat and increasing fatty acid oxidation; stimulation of serum vitamin D_3_ release, prevention against and adaptation to oxidative stress; decrease in chronic inflammation (decrease in cytokine IL-6 and TNF-α); improvement of immune system function; effects on skin moisturisation and physical barriers against pathogens [39,40,50].

Considering the literature data as well as the results of our study, a beneficial effect of vitamin D_3_ supplementation on the severity of psoriasis should be expected. Numerous randomised trials have been published to date evaluating the effectiveness of vitamin D_3_ supplements in controlling the severity of psoriasis. In one recent study conducted in Asia, vitamin D_3_ supplementation at a dose of 60,000 IU once every two weeks was associated with improved skin lesions in patients with psoriasis and reduced PASI scores [51]. A study by Finamor et al. [52] assessed the effect of vitamin D_3_ supplementation at a dose of 35,000 IU daily for six months on the course of psoriasis and vitiligo, and found clinical improvement in the majority of patients, without side effects such as hypercalcemia, hypercalciuria, nephrolithiasis, or nephrocalcinosis. Ingram et al. [53] found no effect of vitamin D_3_ supplementation at a dose of 100,000 IU per month on PASI score improvement compared to placebo. A significant limitation of the latter study, however, was the relatively small number of patients with psoriasis. The lack of improvement in PASI scores after 6 months of oral vitamin D_3_ supplementation was reported in a meta-analysis by Theodoridis et al. [54]. This meta-analysis included only five studies, which were characterised by a high risk of bias in PASI score assessment, and the authors recommended the need for further prospective studies. Also, a meta-analysis of four RCTs reported by Formisano et al. [2] showed that oral vitamin D supplementation did not seem to improve clinical manifestations in patients with psoriasis (*n* = 173).

In our study we found no significant difference in the severity of psoriasis between patients with normal vitamin D_3_ levels (>30 ng/mL) and those with suboptimal levels (20–30 ng/mL). A plausible explanation for this observation is that in most of the patients with normal vitamin D_3_ levels, only a marginal increase was found beyond the lower limit of the optimal range (30–50 ng/mL). The absence of statistical significance may be attributable to the absence of therapeutic benefits resulting from a marginal increase in vitamin D_3_ level above 30 ng/mL. Interestingly, the Polish Society of Endocrinology defines vitamin D_3_ levels in the range of 20–30 ng/mL to be suboptimal, but there is still an ongoing debate regarding the adequate norm for vitamin D_3_ levels [55]. A statement from the Third International Conference on Controversies in Vitamin D concluded that some patients may fully benefit from serum vitamin D_3_ levels in the range of 20–30 ng/mL, but no clear benefit from higher levels has been proven. Following this statement, there is a consensus on two points: vitamin D_3_ levels below 12 ng/mL are clearly deficient, and levels above 30 ng/mL are clearly sufficient [56].

Interestingly, our study found no differences in the severity of psoriasis between patients with optimal vitamin D_3_ levels (>30 ng/mL) and the group of patients with vitamin D_3_ deficiency (<10 ng/mL). The simplest explanation appears to be the small size of the analysed groups, which meant that statistical significance could not be achieved. However, it should be noted that some studies addressing this problem have shown some negative effects of higher vitamin D_3_ levels in patients with chronic diseases. For example, Kuang et al. [57] reported the lowest risk of psoriasis in patients with vitamin D_3_ levels in the range from 10 to 20 nmol/L. Amrein et al. [58] showed that high vitamin D_3_ levels were associated with higher all-cause mortality in hospitalised patients. An attempt to explain this controversy in vitamin D research was made by Kojima et al. [59], who demonstrated that vitamin D supplement users who were the most frail (had the highest number of chronic diseases and severe flare-ups of a chronic disease), significantly more closely adhered to supplementation guidelines and were closely monitored by their physicians in this respect. This likely explains the results obtained in the present study. However, to verify this hypothesis, long-term prospective studies should be conducted with at least several measurements of vitamin D_3_ level, taking into account not only the influence of diet, but also physical activity.

The consensus of experts from Central and Eastern Europe on supplementation guidance for vitamin D recommends the intake of vitamin D for all individuals during the autumn and winter months [60]. It would be particularly worthwhile to adopt vitamin D_3_ supplementation guidelines dedicated to patients with psoriasis, based on the assessment of serum vitamin D_3_ levels. However, economic reasons significantly limit this type of approach, and therefore supplementation in standard doses is recommended, in accordance with the updated guidelines of international scientific associations.

There are many publications regarding the relationship between serum vitamin D_3_ levels and the risk of infections caused by *Candida* spp. For example, Lim et al. [61] demonstrated beneficial effects of higher vitamin D_3_ doses in patients with candidemia caused by *Candida albicans*. The hypothesis on a positive effect of vitamin D_3_ was also supported by Xie et al. [62]. Their study analysed a population from a paediatric intensive care unit and revealed a significant decrease in the prevalence of *Candida* infections in patients receiving a yoghurt drink supplemented with vitamin D_3_. Furthermore, the hospital stay was shorter in these patients. This is important in the context of the ongoing increase in antifungal resistance. Allemailem [63] described a higher efficacy of fluconazole with the addition of vitamin D_3_ in a murine model of candidiasis. The studies presented above provide strong evidence for the antifungal effect of vitamin D_3_.

In our study, no such relationship was observed. This is in line with findings by Muhvić-Urek et al. [64]. These authors analysed the association between vitamin D_3_ levels and *Candida*-associated denture stomatitis. Cholecalciferol deficiency had no significant effect on the frequency of *Candida*-associated stomatitis. However, it should be emphasised that our study covered a small population of patients. It is possible that many of the factors described above which influence vitamin D_3_ levels and oral *Candida* spp. colonisation have made the effect of vitamin D_3_ on colonisation to be negligible in clinical conditions. The effects of vitamin D_3_ could reach statistical significance in a very large population.

The clinical manifestation of psoriasis in the oral cavity remains poorly understood [65], and the studies based on clinical practice are still limited. Using quantitative analysis of immunoexpression of selected inflammatory markers in epithelial cells of the oral mucosa, we observed a significantly higher percentage of cells expressing the IL-12, IL-17, and TNFα cytokines in patients with severe to moderate psoriasis and in patients with mild psoriasis compared to healthy controls. In patients with severe to moderate psoriasis, the percentage of cells expressing IL-12 was also significantly higher compared to subjects with mild disease. These findings are consistent with the concept that psoriasis is driven by a proinflammatory cytokine network [1,2,4,5].

The increased expression of proinflammatory cytokines observed in the oral epithelium suggests the presence of local inflammatory processes within the oral mucosa. These findings may indicate that the inflammatory response in psoriasis is not confined to the skin; however, since cytokine expression was not assessed in serum or in psoriatic skin lesions in parallel, our data do not allow direct conclusions regarding the systemic nature of the inflammation. It should be noted, however, that psoriatic lesions in the oral cavity are rarely detected in clinical practice. This may be due to a lack of thorough assessment of the mouth during routine clinical examination or to the nonspecific clinical picture of mucosal psoriasis. Furthermore, the epidermal renewal cycle in psoriasis is accelerated to the length of the epithelial renewal cycle in the oral mucosa [66]. Therefore, it can be assumed that oral lesions may be subclinical and imperceptible in macroscopic inspection. These observations suggest that patients with psoriasis may experience a low-grade inflammatory response within the oral cavity. This is in line with the findings of Ganzetti et al. [67], who reported increased levels of proinflammatory TNF-α in the saliva of patients with psoriasis compared with healthy controls.

The present study has several limitations. The relatively small sample size may have reduced the statistical power of some analyses. In addition, several potential confounding factors associated with both vitamin D_3_ status and psoriasis severity, including age, sex, smoking habits, physical activity, dietary factors, and concomitant disorders, were not included in the statistical models. Oral *Candida* colonisation was assessed using oral swabs, which may be less sensitive than oral rinse methods. Moreover, cytokine expression was evaluated exclusively in oral epithelial cells, without parallel assessment of serum cytokine concentrations or psoriatic skin lesions. Future studies should include larger cohorts and a more comprehensive adjustment for potential confounding variables.

This study also has strengths because it presents a comprehensive measure of the relationship between oral *Candida* spp. colonisation, serum vitamin D_3_ concentration, cytokines expression and psoriasis severity.

## 5. Conclusions

This research used a comprehensive measure of the role of vitamin D_3_ serum levels, oral mucosal colonisation by *Candida* spp., and immunoexpression of inflammatory markers IL-12, IL-17, and TNF-α in patients with psoriasis. In doing so, there were comparisons made with a control group. The results of this study suggest a possible association between lower vitamin D_3_ serum levels and greater psoriasis severity. However, this relationship was not consistently observed across all analyses. A statistically significant association was identified when disease severity was assessed using BSA scores, whereas PASI scores and categorical analyses demonstrated only statistical trends. This is consistent with many previous reports, which implicated low vitamin D_3_ levels as an important risk factor for psoriasis. Although the exact role of vitamin D in psoriasis pathogenesis is not clearly explained, data indicate that vitamin D_3_ deficiency is associated with a microbiological imbalance accompanied by *Candida* spp. infection. Our results did not show a significant difference in the oral mucosa colonisation by *Candida* spp. between patients with psoriasis and controls, regardless of vitamin D_3_ levels. When examining the influence of vitamin D_3_ levels and *Candida* spp. colonisation of the oral mucosa, we found no interaction between these variables. In turn, examining the effect of these variables on psoriasis severity, we found a trend toward a positive association between higher vitamin D_3_ levels and the absence of *Candida* spp. interaction in relation to psoriasis severity.

Further investigation revealed significantly higher immunoexpression of the inflammatory markers IL-12, IL-17, and TNF-α in the oral mucosa of patients compared with controls. The expression of IL-12 was higher in patients with moderate or severe psoriasis than in patients with mild psoriasis. The percentage of oral mucosal cells expressing TNF-α was higher only in patients with severe or moderate psoriasis. Further prospective studies, conducted in larger cohorts and employing more sensitive detection methods, are needed to determine whether vitamin D_3_ deficiency contributes directly to psoriasis severity and whether it influences the microbiological environment of the oral cavity. Thus, our findings indicate the presence of a local inflammatory response within the oral mucosa of patients with psoriasis.

Despite growing scientific evidence and knowledge of the leading role of proinflammatory cytokines in the pathogenesis of psoriasis, the exact mechanisms by which these cytokines play an essential role in the disease process remain unclear, due to the complexity of the association and its multifactorial nature. To obtain more specific results, further studies with a large sample size are needed, along with a complete assessment of modifiable lifestyle-related factors to enhance statistical power and define the optimal serum vitamin D levels for psoriasis. The studies should include participation of multidisciplinary health specialists. This approach could clarify existing cellular mechanisms and discover new strategies to prevent excessive proinflammatory cytokines production.

## Figures and Tables

**Table 1:** Basic characteristics of demographic variables, BMI and the period of vitamin D_3_ intake in patients with psoriasis and controls.

	Patients with Psoriasis *n* = 59	Controls *n* = 32	*t*/χ^2^	*p*
Age (years): M (SD)	48.03 (14.60)	47.78 (14.89)	0.08 ^a^	0.938
Gender: female/male	18/41	20/12	8.73 ^b^	0.003
Body weight (kg): M (SD)	88.18 (19.42)	84.78 (24.38)	0.73 ^a^	0.470
Body height (m): M (SD)	1.73 (0.08)	1.71 (0.08)	1.37 ^a^	0.173
BMI: M (SD)	29.31 (5.65)	28.88 (6.97)	0.32 ^a^	0.752
Period of vitamin D_3_ intake: September–March/April–August	45/14	25/7	0.04 ^b^	0.841

M (SD)—mean ± standard deviation; BMI—Body Mass Index; ^a^ parametric Student’s *t*-test; ^b^ chi-square test for cross-tabulation.

**Table 2:** Basic characteristics of demographic and clinical variables for patients with different severities of psoriasis and controls.

Characteristic	Patients with Psoriasis		Controls *n* = 32	*F*/χ^2^	*p*
	Severe to Moderate Psoriasis *n* = 25	Mild Psoriasis *n* = 34			
Age (years): M (SD)	45.88 (13.99)	49.62 (15.04)	47.78 (14.89)	0.47 ^a^	0.628
Gender: female/male	3/22	15/19	20/12	14.84 ^b^	0.000
Body weight (kg): M (SD)	88.67 (19.94)	87.84 (19.34)	84.78 (24.38)	0.27 ^a^	0.764
Body height (m): M (SD)	1.76 (0.08)	1.71 (0.08)	1.71 (0.08)	2.96 ^a^	0.057
BMI: M (SD)	28.65 (5.68)	29.78 (5.66)	28.88 (6.97)	0.28 ^a^	0.753
Period of vitamin D_3_ intake: September–March/April–August	19/6	26/8	27/7	0.04 ^b^	0.979

M (SD)—mean ± standard deviation; BMI—Body Mass Index; ^a^ parametric one-way ANOVA F-test; ^b^ chi-square test for cross-tabulation.

**Table 3:** Basic characteristics of demographic and clinical variables for patients with psoriasis and different levels of vitamin D_3_.

Characteristic	Patients with Psoriasis			*F*/χ^2^	*p*
	Low Level of Vitamin D_3_ *n* = 32	Suboptimal Level of Vitamin D_3_ *n* = 13	Optimal and High Levels of Vitamin D_3_ *n* = 14		
Age (years): M (SD)	44.19 (14.49)	55.77 (13.66)	49.64 (13.39)	3.25 ^a^	0.046
Gender: female/male	7/25	8/5	3/11	7.57 ^b^	0.023
Body weight (kg): M (SD)	90.74 (20.03)	84.38 (17.31)	86.04 (20.38)	0.60 ^a^	0.555
Body height (m): M (SD)	1.76 (0.08)	1.67 (0.07)	1.74 (0.07)	6.93 ^a^	0.002
BMI: M (SD)	29.28 (5.36)	30.39 (5.91)	28.38 (6.26)	0.42 ^a^	0.662
Period of vitamin D_3_ intake: September–March/April–August	26/6	9/4	10/4	0.98 ^b^	0.614

M (SD)—mean ± standard deviation; BMI—Body Mass Index; ^a^ parametric one-way ANOVA F-test; ^b^ chi-square test for cross-tabulation.

**Table 4:** Prevalence of oral *Candida* colonisation.

Culture for *Candida* spp.	Patients with Psoriasis	Controls	χ^2^	*p*
	Number of Subjects (*n*)			
Positive	29	16	0.010	0.938
Negative	30	16		
	Patients with moderate to severe psoriasis	Patients with mild psoriasis		
Positive	12	17	0.02	0.0879
Negative	13	17		

**Table 5:** Effect of vitamin D_3_ serum levels on the severity of psoriasis.

Severity of Psoriasis	Patients with Low Vitamin D_3_ Level	Patients with Suboptimal Vitamin D_3_ Level	Patients with Optimal or High Vitamin D_3_ Level	χ^2^	*p*
	Number of Patients (*n*)				
Moderate to severe	17	2	6	5.39	0.067
Mild	15	11	8		

**Table 6:** Effect of vitamin D_3_ serum levels on the severity of psoriasis measured with PASI and BSA.

Clinical Tool for Measuring the Severity of Psoriasis	Patients with Low Vitamin D_3_ Level M (SD)	Patients with Suboptimal Vitamin D_3_ Level M (SD)	Patients with Optimal or High Vitamin D_3_ Level M (SD)	*F*	*p*	*ɳ* ^2^
PASI score	11.23 (11.04)	3.55 (3.76)	8.34 (7.91)	3.10	0.053	0.10
BSA score	27.77 (27.01)	6.23 (7.13)	21.21 (22.70)	3.84	0.028	0.13

PASI—Psoriasis Area Severity Index, BSA—Body Surface Area; M—mean ± standard deviation.

**Table 7:** Effect of vitamin D_3_ serum levels on oral *Candida* colonisation in the studied population.

Culture for *Candida* spp.	Vitamin D_3_ Level			χ^2^	*p*
	Low	Suboptimal	Optimal or High		
	Patients with Psoriasis *n* = 59				
Positive	19	6	4	3.76	0.153
Negative	13	7	10		
	Controls *n* = 39				
Positive	4	4	8	2.26	0.323
Negative	7	5	4		

**Table 8:** Effect of different vitamin D_3_ serum levels and the presence or absence of oral *Candida* colonisation on the severity of psoriasis measured with PASI.

	Patients with Low Vitamin D_3_ Level M (SD) [CI]	Patients with Suboptimal Vitamin D_3_ Level M (SD) [CI]	Patients with Optimal or High Vitamin D_3_ Levels M (SD) [CI]	*F*	*p*	*ɳ* ^2^
Positive culture for *Candida* spp.	12.93 (14.03) [6.17–19.69]	4.10 (4.01) [−0.11–8.31]	9.48 (6.73) [−1.23–20.19]	Effect size of vitamin D_3_ level		
				3.14	0.051	0.11
Negative culture for *Candida* spp.	11.12 (9.13) [5.60–16.64]	3.09 (3.78) [−0.41–6.59]	7.88 (8.63) [1.71–14.05]	Effect size of colonisation with *Candida* spp.		
				0.24	0.628	0.00
Interaction between vitamin D_3_ level and *Candida* colonisation				Effect size of interaction between vitamin D_3_ level and colonisation with *Candida* spp.		
				0.01	0.993	0.00

PASI—Psoriasis Area Severity Index; M—mean ± standard deviation; CI—95% confidence interval.

**Table 9:** Significance of differences between patients with psoriasis and different vitamin D_3_ levels, in terms of the presence or absence of oral *Candida* colonisation, and in terms of the severity of psoriasis measured with BSA.

	Patients with Low Vitamin D_3_ Level M (SD) [CI]	Patients with Suboptimal Vitamin D_3_ Level M (SD) [CI]	Patients with Optimal or High Vitamin D_3_ Levels M (SD) [CI]	*F*	*p*	*ɳ* ^2^
Positive culture for *Candida* spp.	27.84 (28.60) [14.05–41.63]	8.00 (5.97) [1.73–14.27]	28.25 (25.98) [−13.10–69.60]	Effect size of vitamin D_3_ level		
				4.09	0.022	0.13
Negative culture for *Candida* spp.	29.38 (25.22) [14.13–44.63]	4.71 (8.14) [−2.82–12.24]	18.40 (22.11) [2.58–34.22]	Effect size of colonisation with *Candida* spp.		
				0.31	0.582	<0.01
Interaction between vitamin D_3_ level and colonisation with *Candida* spp.				Effect size of interaction between vitamin D_3_ level and colonisation with *Candida* spp.		
				0.25	0.779	<0.01

BSA—Body Surface Area; M—mean ± standard deviation; CI—95% confidence interval.

## Data Availability

The original contributions presented in this study are included in the article. Further inquiries can be directed to the corresponding author.

## Abbreviations

The following abbreviations are used in this manuscript:

AMPs	antimicrobial peptides
BMI	Body Mass Index
BSA	Body Surface Area Index
IFN-γ	interferon gamma
IL12	interleukin 12
IL17	interleukin 17
M	mean
MCF	McFarland scale
NETs	Neutrophil Extracellular Traps
PASI	Psoriasis Area and Severity Index
SD	standard deviation
TLRs	toll-like receptors
TNFα	tumour necrosis factor alpha
UVB	ultraviolet B
